# Food insecurity, xenophobia, and political legitimacy: exploring the links in post‐COVID‐19 South Africa

**DOI:** 10.1111/disa.12667

**Published:** 2024-11-07

**Authors:** Khulekani T. Dlamini, Elizabeth Hull

**Affiliations:** ^1^ Independent Researcher and Translator South Africa; ^2^ SOAS University of London United Kingdom; ^3^ Swedish Collegium for Advanced Study Sweden

**Keywords:** COVID‐19, food insecurity, food prices, governance, political legitimacy, rural food systems, South Africa, xenophobia

## Abstract

Food insecurity in South Africa was critical prior to the COVID‐19 outbreak, but the problem deepened quickly during the pandemic when government controls caused job losses, a food supply collapse, and escalating hunger. The food and fuel price hikes and political instability that followed led to the July 2021 ‘unrest’, which left more than 350 people dead. Behind this lay a crisis within the governing African National Congress. In this paper, we draw on in‐depth interviews and ethnography with individuals working in food‐based livelihoods to investigate how people continued to secure food, and how rural food systems were affected. Against a backdrop of hunger, social unrest, and xenophobic hostility, we consider how people perceive the state in a rural area of KwaZulu‐Natal. We argue that weak governing institutions and South Africa's exposure to globally‐triggered spikes in food and fuel prices are leading to food insecurity. Hunger, in turn, is contributing to a crisis of legitimation for the state.

## 1 INTRODUCTION

In 2020, government controls to curb the transmission of COVID‐19 in South Africa caused huge job losses and food insecurity. During the first ‘lockdown’, three million people lost their livelihoods and 47 per cent of households ran out of money to buy food (Bridgman, van der Berg, and Patel, 2020). The situation prior to the lockdown was already critical, with a stunting rate of 27 per cent in children under five (National Department of Health et al., 2019, p. 11). But hunger escalated rapidly during the lockdown, exacerbated by the rising costs of food and fuel and other disturbances such as social unrest and electricity blackouts. This was happening in the context of growing xenophobia, often targeting small food retailers, who became easy scapegoats as the government failed to deliver on the population's constitutionally‐enshrined right to food.

This paper situates food insecurity in South Africa in the realm of wider political tensions. Drawing on in‐depth interviews and ethnography with people with food‐based livelihoods in northern KwaZulu‐Natal province, we investigate two key issues. First, how did people navigate food insecurity and how have rural food systems been affected during the turbulent period of COVID‐19 and subsequently? Second, how is food insecurity contributing to a crisis of legitimacy for the governing African National Congress (ANC)? We show that food insecurity is linked both to xenophobic attitudes and to a collapse in support for the ANC. However, experiences vary along the lines of gender, class, age, and citizenship status, defying singular explanations.

At the beginning of the COVID‐19 emergency, the South African government reacted swiftly, imposing a national ‘lockdown’ that lasted for three weeks. As in many parts of the world, its aim was to keep people in place to reduce transmission of COVID‐19. Eager to distance itself from the (Jair) Bolsonaro‐style indifference of the Brazilian response, South Africa's lockdown approach sought to enhance its global reputation for responsible governance (Bank and Sharpley, 2022, p. 13). Yet, the government's actions turned out to be ‘disastrous’ according to the sociologist Jeremy Seekings (2020): it had no plan for mitigating food insecurity when livelihoods and incomes collapsed. This not only produced a food insecurity crisis but ultimately failed to prevent widespread transmission. The COVID‐19 death toll in South Africa was significantly higher than in any other country on the continent (Green, 2022).

Several extensions to South Africa's social protection system were ushered in to stave off destitution and hunger, including supplements to existing social grants. The percentage of the population receiving grants rose from 31 per cent before the pandemic to more than 47 per cent by 2023 (SASSA, 2023, p. 9). These measures were essential but they were beset with bureaucratic hurdles and delays. Grants did not reach many eligible recipients on time or in some cases at all. The government also provided free food parcels, but many people received them only once or not at all despite being eligible. Such feeding schemes were ‘a tiny fraction of what was needed urgently’, wrote Seekings (2020, p. 1), adding that governmental distribution of free food declined significantly overall when taking into account the closure of the National School Nutrition Programme that feeds some nine million children daily. As communities and civil society organisations stepped in to feed people, commentators described local ‘acts of kindness’ while ‘politicians floundered’ (Jahajeeah, 2021).

Social tensions reached a climax in July 2021 when violence erupted. Rioting and looting of shops began in KwaZulu‐Natal and quickly spread elsewhere. Sparked by the imprisonment of former President Jacob Zuma, commentators recognised that COVID‐related job losses, economic insecurity, and hunger motivated the riots (Hunter, Wicks, and Singh, 2021). The crisis began with inflammatory social media comments threatening unrest should Zuma remain in jail. He had received a 15‐month sentence for failing to cooperate with an investigation into corruption during his time as president. Many believe that his close supporters orchestrated organised acts of sabotage, arson, and looting in various parts of the coastal city of Durban.

The riots spread quickly to other parts of KwaZulu‐Natal and to Gauteng, South Africa's most populous province, where Johannesburg is located. They persisted for several days until the deployment of some 25,000 soldiers, alongside overwhelmed police forces, restored order. Speculation arose about possible police involvement in the unrest, fuelled by suspicions of corruption within the police force under the previous Zuma leadership. These security concerns left many feeling vulnerable and unprotected amidst the chaos.

More than 350 people were killed. In KwaZulu‐Natal, 200‐plus shopping centres suffered looting or damage, while 100 malls were subject to arson attacks. Overall, 40,000 businesses and 50,000 traders were affected, while stock worth ZAR 1.5 billion was lost and 150,000 jobs were estimated to be at risk (van Zyl, 2021). Food supply was curtailed dramatically in the immediate aftermath.

Since then, defaulting energy services and rising prices have ensured that tensions remain high. Corruption scandals and management failings of the state‐owned energy provider, Eskom, led to blackouts of up to nine hours per day in 2023—typically referred to as load‐shedding—disrupting livelihoods on a large scale. Electricity cut‐outs damaged and slowed down food supply chains (SCN Africa, 2023). Moreover, the country has experienced escalating prices for food and fuel.^1^ These changes have put pressure not only on household food security but also on the rural food economy, where sellers face steep competition from supermarkets.

In section two, we set out a theoretical framework for understanding the connections between food security and political instability, drawing on Gillian Hart's (2014) concepts of de‐ and re‐nationalisation. In section three, we explain the context for the research and the methods. In section four, we describe people's encounters with the state as they try to mitigate food insecurity, including the vulnerabilities experienced by migrants. In section five, we discuss the pressures exerted on the rural food system precipitated by the COVID‐19 lockdown and subsequent events. In section six, we reflect on people's perspectives of the South African state and their expectations of it. Memories and explanations of the July ‘unrest’ were a pertinent lens for exploring questions of state legitimacy at a time of extreme food insecurity. The riots were a moment when connections between food insecurity, xenophobia, and state legitimacy were laid bare. Yet, our data also suggest caution about singular explanations of why people rioted. Political subjectivities interacted with personal experience to produce a diverse picture. The paper ends (section seven) with some conclusions.

## 2 THE POLITICS OF FOOD SECURITY THROUGH THE LENS OF DE‐ AND RE‐NATIONALISATION

In August 2012, police opened fire and killed 34 striking miners close to the town of Marikana near Rustenburg in South Africa's North West province, in a defining moment for the country. Hart (2014) interpreted this event as a consequence of a protracted crisis arising from contradictory processes of de‐ and re‐nationalisation. In the general election on 29 May 2024, almost 12 years after the Marikana shooting, the ANC lost its majority rule for the first time since the beginning of democracy 30 years prior. This weakening state legitimacy in the context of growing hunger and widespread unemployment reveals the contradictions between de‐ and re‐nationalisation emerging again in dramatic and urgent ways. In this section, we explain these concepts and their usefulness for understanding links between food insecurity and political fragility.

According to Hart (2014), South Africa's entry into the financialised global economy, the entrenchment of white corporate interests, and large‐scale capital flight were hallmarks of ‘de‐nationalisation’. Recognisable features of global neoliberalism interacted with South Africa's racialised legacies of settler‐colonialism and apartheid to produce deepening inequalities and surplus populations, leading to an untenable situation of poor wages and conditions among the miners.

Pushing in the opposite direction, Hart (2014) used the term ‘re‐nationalisation’ to describe the ideological terrain of nationhood in post‐apartheid South Africa. This included attempts to unify the nation—with the ANC at its centre—through official articulations of nationalism and claims to moral authority based on the party's origins as a liberation movement. Central to processes of re‐nationalisation also were anti‐immigrant policies and rhetoric alongside popular vigilantism, resulting in sometimes lethal xenophobic outbursts. Hart (2014) suggested that these processes are in tension with one another, giving rise to populist, nationalist, and xenophobic politics. The shooting of the miners in Marikana, for Hart, was a sign of the losing grip of the ANC's hegemony, as it tried both to defend international corporate interests and to contain protest and dissent.

These contradictory processes are apparent in current political tensions around food provisioning. Rapidly escalating food insecurity is a consequence of policies of de‐nationalisation. Like many governments, the ANC has embraced the prevailing economic paradigm of globalisation—the deregulated, debt‐driven, export‐oriented ‘growth’ model, which suppresses domestic wages while amplifying the profits of exporters. The corresponding rise of corporate retail meant that when COVID‐19 hit, the government leant heavily on supermarkets for delivering food security while leaving informal retailers to navigate the situation alone (Battersby, 2020; Hull and Masinga, 2023). De‐nationalisation has also made South Africa's food system susceptible to abrupt, globally‐triggered spikes in food and fuel prices. For example, the Russia–Ukraine war reduced global wheat supply, pushing up prices of the staple commodity. Moreover, global financial speculators reacted negatively to President Cyril Ramaphosa's political non‐alignment with United States foreign policy on the war, causing the South African rand to fall against the dollar (Pettifor, 2023). The price of imports increased further, and South Africans faced rocketing fuel and food prices.^2^

Processes of re‐nationalisation, too, intensified in the lead up to the May election. Xenophobic rhetoric featured in attempts by ANC factions and competing parties to garner support. Frequently, the target was foreign‐owned food retailers. In a visit to Bushbuckridge in November 2023, Minister of Home Affairs Aaron Motsoaledi made bombastic statements about deporting foreign owners of unregistered shops (SABC, 2023). This occurred months after the anti‐immigrant vigilante group Operation Dudula stormed into foreign‐run spaza shops (small consumer goods retailers), harassing shopkeepers and threating closure (Schwikowski, 2023). In March 2024, former South African President and leader of the newly‐formed uMkhonto weSizwe (MK) party, Jacob Zuma, released a video on TikTok stating that there was ‘no crime’ in South Africa ‘before foreign nationals came’ (Pilling and Mark, 2024).

The rural food economy is shaped by these contradictory forces of de‐ and re‐nationalisation, culminating in the social unrest of 2021. We explore these dynamics in relation to the challenges faced by those working in food‐based livelihoods. To what extent are the ‘politics of provisions’ (Scott‐Villiers and Hossain, 2017) linked to the re‐nationalising logics of xenophobia? How are expectations of the state and its legitimacy shaped by struggles for food and livelihood?

## 3 CONTEXT AND METHODS

This paper is based on research in uMkhanyakude District Municipality in KwaZulu‐Natal, a predominantly rural municipality in the far north of the province, sharing borders with Mozambique and Eswatini. It is one of South Africa's most deprived municipalities, with extremely high levels of household food insecurity and unemployment (Nhlozi, 2023). In surveys conducted during the COVID‐19 pandemic, 70 per cent of households were food‐insecure in KwaZulu‐Natal (Simelane et al., 2023, p. 14), as compared to 64 per cent of households nationally (Simelane et al., 2024, p. 16). In KwaZulu‐Natal, 16 and 4 per cent of households experienced moderate hunger and severe hunger, respectively (Simelane et al., 2023, pp. 14–15). Survey evidence from uMkhanyakude showed even more severe food insecurity, scoring lower than provincial averages for most food security and nutrition indicators (Simelane et al., 2023, p. 16). Across regional and national surveys, food insecurity has deepened over the past decade.

The study is not intended to be representative of South Africa as a whole, but instead provides a case study of one rural region impacted by the 2021 riots: the town of Jozini and its surrounding hinterland in northern KwaZulu‐Natal. The region is a pertinent location for the analysis given both its high rates of food insecurity and political fragility, judging by social unrest and high levels of voter dissatisfaction. Commentators have often regarded KwaZulu‐Natal as politically exceptional, with its rural populations never voting consistently for the ANC, as in other rural parts of South Africa. After the riots this perception intensified, described increasingly in terms of a ‘KwaZulu‐Natal problem’ (Buccus, 2021). These narratives recognised the province not only as the centre of the recent riots, but also as the stage for intense power struggles within the governing ANC between two factions represented by President Ramaphosa and former President Zuma, with the latter garnering much support in his home province of KwaZulu‐Natal. The province's deeper history of Zulu ethnic nationalism reappeared in these contestations. Fighting between the ANC and the Zulu nationalist Inkatha Freedom Party (IFP) in the early 1990s threatened to derail the country's impending liberation from apartheid rule. Subsequently, the ANC has never achieved the same support in the province as elsewhere. This was revealed starkly in the general election on 29 May 2024, in which Zuma's new populist MK party captured 45 per cent of the vote in comparison to the ANC's 17 per cent, down from 54 per cent in 2019.^3^ The links between food security and state fragility are refracted through these local politics. This in turn contributes to the wider, shifting ‘politics of provision’ nationally, referring to the ways that people and rulers interact over struggles for subsistence (Scott‐Villiers and Hossain, 2017).

This paper draws on qualitative methods including ethnography and in‐depth interviews. While quantitative data from surveys have revealed the scale of the hunger crisis in South Africa, in‐depth research is needed to shed light on how people are responding and adapting. By privileging context and voice, these methods are best placed to reveal experiences not currently visible to state and development agencies. Their value is not in producing generalisable knowledge, but rather in providing insight into context‐embedded meanings and in generating data amenable to comparison with regions undergoing similar processes. Individual testimonies serve as illustrations of prominent themes identified in the data, while also elucidating personal experiences that general statements fail to capture. The value of this, in part, is to bridge the information divide between policymakers and academics on the one hand, and marginalised, rural populations on the other.

We use several data sources in the paper. First, we draw on in‐depth interviews undertaken between 2022 and 2024 with 40 people working in food‐related livelihoods, including farmers, street vendors, tuck shop owners, supermarket employees, and people engaging in collective food acquisition strategies, for example, as members of *stokvels* (rotating savings clubs). Interviews lasted between one and two hours. We aimed to find out how people understood and navigated recent disruptions, including COVID‐19, price rises, and the July 2021 ‘unrest’, in terms of meeting their own food security and livelihood needs. We also sought to determine what their experiences might reveal about the vulnerabilities or continuities of rural food systems during these shocks, as well as the impact on perceptions and expectations of the state. We combined purposive and convenience sampling to select participants, the latter because we have been conducting ethnographic research in the area since 2010; hence, we have deep knowledge of the context serving as a basis for meaningful research.

Some of the research participants we have known for several years owing to long‐term ethnographic research. Therefore, we also draw, secondly, on ethnographic data, enabling interpretive depth based on long‐term research engagement. Given the practical and ethical obstacles to conducting ethnographic research during and after the COVID‐19 pandemic, there is a lacuna of such work during this period in South Africa and elsewhere. We developed a collaborative approach to data gathering in the context of these constraints and rely on interviews with research participants for understanding people's experiences in this recent time frame.

The paper also draws on a report of a pilot project conducted by Elizabeth Hull, involving three community researchers in the same area, Khulekani T. Dlamini, Cynthia Gina, and Simangaliso Masinga. The report explores community responses to declining food access after COVID‐19 in South Africa (Hull et al., 2023).

Where relevant, we situate our findings comparatively in relation to national‐level debates based on secondary reading. Khulekani T. Dlamini, a trained translator and interpreter who grew up and lives near the research site, carried out the interviews. He translated and transcribed them and produced preliminary written interpretations. For interviews and ethnographic data, Hull performed initial coding using NVivo 12 qualitative data analysis software. The two authors collaborated on developing analysis and interpretations of the data. Hull wrote the paper, incorporating Dlamini's edits and comments on several drafts. We discuss our collaborative approach in more detail elsewhere (Dlamini and Hull, 2024).

Research participants encompassed foreign and South African migrants as well as people who were born in the area. Almost everyone included in the study, apart from migrants, depended to varying degrees on government protection in the form of a pension, child support grant, disability grant, or the recently‐introduced COVID‐19 Social Relief of Distress (SRD) grant, or belonged to a household where other members depended on it. Some benefited from secure access to land or well‐established supply chains, whereas others depended on short‐term land access or erratic networks to source, transport, or sell products. Many pursued informal work within the ‘survivalist’ or farming sectors, growing, procuring, or selling very small quantities of produce. Five respondents were tuck shop owners and nine worked in formal sector employment, such as in supermarkets or fast‐food establishments, exclusively or in addition to other income‐generating activities. As is well‐documented in the anthropological literature on livelihoods in South Africa, people typically sought to establish multiple income streams if they had the capacity (Francis, 2000; Neves and du Toit, 2013; Hull, 2017, pp. 75–76). This was a way of making money go further and of spreading risk should one income source lapse.

## 4 FOOD INSECURITY AND STATE INTERACTIONS

In this section, we explore how experiences of food security are mediated by encounters with the state. We show that despite high levels of reliance on government grants and other forms of government assistance, this support is experienced unevenly, depending on citizenship status, bureaucratic hurdles, and perceptions of corruption. Its effectiveness is also undermined by high food prices, producing ambivalence and mistrust in government.

### 4.1 Citizenship status

Across all of our data, those facing precarious or ‘survivalist’ livelihoods struggled to obtain enough food for themselves and their families. The lockdown was an extreme time for these people, who faced acute hunger and used strategies such as reducing the meal size of adults to ensure children were fed. Hunger was frequently severe among migrants who had no recourse to government support during the lockdown. One participant described having nothing in their room to consume but water and another skipped meals so that their one‐year‐old child could eat. Attempts to claim government support, either on legitimate or fraudulent grounds, evoked fear of arrest or deportation. This was exacerbated by concerns about rising xenophobia. For example, two foreign street vendors said that they were anxious about government reprisal after hearing that South African vendors were lobbying the local municipality to prevent migrants from operating. This supports research findings from other regions, such as Cape Town, where migrants faced the ‘double jeopardy’ of lacking government support while also being vilified as undesirable ‘outsiders’ (Crush and Tawodzera, 2024). While South African interlocutors expressed a wide range of views, we also identified xenophobic sentiments often linked to personal experiences of hardship, such as: ‘Should we die of hunger in our own land and let migrants live better in a foreign land?’

### 4.2 Bureaucratic encounters with the state

The SRD grant of ZAR 350 was widely received. It was disbursed to recipients' bank accounts or collected from supermarkets. Interviews reflected widespread recognition that this extension of state support was essential yet inadequate for meeting households' food needs. Like elsewhere in the country, bureaucratic obstacles meant that some who were eligible did not receive it (Kajiita and Kang'ethe, 2024, p. 1139). Moreover, the positive impact of the grant was undermined by rising food prices. Several interlocutors viewed this in terms of a democratic deficit since for them, it revealed corporate collusion by the government with supermarkets, which were the true recipients of the money owing to price hikes. This reinforced a belief that their needs were the lowest priority of the government (Hull et al., 2023). There were also reports of people arriving at a supermarket only to find their grant unavailable, leading to rumours that government officials or supermarkets were siphoning off the money.

Bureaucratic hurdles influenced perceptions of state legitimacy. Administrative failings and perceptions of corruption were closely linked. For example, one respondent registered for a food parcel but never received it, believing that government officials used her details and then kept the food for themselves. Irregular grant payments could also be associated with a perception of an all‐seeing state, collaborating with banks and other corporate actors. For instance, a grant payment was discontinued unexpectedly for reasons unknown to the recipient. She suspected that the government had access to her bank account details and could see that her son was sending remittances from the city. Regardless of the veracity of this claim, it illustrates a perception of close entanglements between politics and the financial system, hinting at the exposure people felt.

### 4.3 The state's absence

While people felt exposed to state surveillance, at other times they felt ignored. A group of farmers told us their livelihoods had ceased for more than six years due to faulty water infrastructure at the irrigation scheme where their fields were located. For them, COVID‐19 was never the problem. Their lifestyles already resembled ‘social distancing’: they worked in the fields and socialised outdoors. Instead, the focus on COVID‐19 meant continuing to be locked out from development efforts. Such testimonies point to deeper frustrations and exclusions rendered invisible by the immediacy of a ‘crisis’. This reflects Janet Roitman's (2013) observation that crisis as a ‘narrative device’ elevates short‐term events to the locus of sociopolitical action, thereby obscuring deeper inequalities. Another example is the highly medicalised response to COVID‐19, focusing on vaccination uptake but leaving families to navigate food insecurity without sufficient support.

As reported by academics and journalists across the country (see, for example, Adelle and Haywood, 2021; Vibert, 2022), people in uMkhanyakude also relied overwhelmingly on collective and mutual assistance at a local scale. There are very few non‐governmental organisations operating in the area, so help came mainly via existing social infrastructures, including family, neighbours, and churches. A school head resumed school meal provision despite the government's discontinuation of the nutrition programme, after quickly realising the scale of hunger that would ensue among children in its absence (Hull et al., 2023). We also recorded piecemeal instances of assistance, such as landlords foregoing rent and a driver cancelling children's school transport fees: situations where failure to pay might have usually resulted in indebtedness or more serious sanctions.

To conclude, we observed that people draw on a range of social infrastructures, both governmental and community‐based, to navigate food insecurity. Failures to access state support can often feel punitive or unfair, fuelling perceptions of corruption, surveillance, or indifference on the part of government. These are linked both to xenophobic sentiments and to perceptions of government collusion with corporations such as banks and supermarkets, demonstrating the logics of both de‐ and re‐nationalisation at play.

## 5 FOOD‐BASED LIVELIHOODS AND THE RURAL FOOD ECONOMY

Although seemingly peripheral to the dominant food system, ‘traditional’ food sector workers form at least 40 per cent of the food market (Bhana, 2018). They are essential for many households' food access strategies, providing food close to homes and offering essential items on credit through long‐term, trust‐based relationships with customers (Hull, 2024). During lockdown, the South African government relied on the corporate food system to meet food security needs, leaving these smaller retailers, farmers, and traders to fend for themselves (Hull and Masinga, 2023). This section describes how people navigated livelihood challenges during and after this period. Prominent themes include: the impact of militarised lockdown enforcement; the vulnerabilities experienced by migrants; the effects of unregulated employment practices in the informal sector; and the shock‐absorbing role of the rural economy.

### 5.1 Militarised lockdown

The government's policy to keep people in place to curb the spread of disease was disastrous for rural livelihoods that frequently depend on mobility. At the beginning of the lockdown, informal enterprises were forced to comply, an approach that softened as it became untenable. As in many other parts of the country, lockdown was enforced through a strong police and military presence (Bank and Sharpley, 2022; Crush and Tawodzera, 2024). Many people wished to comply with it, not least because they feared contracting the virus themselves. However, livelihood necessities compelled people to evade lockdown.

News reports from around the country highlighted the impact on production of lockdown restrictions that prevented farmworkers from travelling (Nobanda, 2020). This was corroborated in our data, which contain examples of small commercial farmers losing entire harvests or suffering crop theft. These problems were exacerbated by the rising costs of inputs such as fertilisers and manure. To recount one experience, a farmer who was renting land for a season recalled being arrested while traveling to the field to change the water sprinklers. She was put into a van full of other detained individuals who had been travelling to work and then held at the police station until the evening: ‘They were verbally abusive and did not want us to call our families. They treated us as serious criminals, but later they allowed us to use our phones’. She subsequently obtained a permit from the police allowing her to travel to the farm, but her employees lacked such documentation. She lost a full two‐hectare crop of maize as a result. At the time of the interview in 2023, she had not yet generated enough income to invest in further production. Reflecting on movement controls enforced by the police and military during COVID‐19, this farmer described how people carried children to the clinics for vaccinations via the bush paths instead of the roads to avoid the punitive actions of lockdown‐enforcing soldiers. She likened the restrictions to those imposed by the apartheid government.

Like farmers, small shop owners and vendors were initially prevented from operating during lockdown. Criminalisation of normal activities posed a particular risk to migrants. One foreign shop owner was arrested temporarily for operating a shop. While he had closed his other two shops, he kept this one open and sold items through a window. He described the hard lockdown as ‘equal to death’, explaining that he had no other source of income. He employed five women informally across his three shops, all of whom he was compelled to lay off. They went to live with family members. Elsewhere, news reports drew attention to police shutting down foreign‐owned spaza shops (Sizani, 2020). This followed a statement by Minister of Small Business Development Khumbudzo Ntshavheni stipulating that only South African‐owned and ‐operated shops would be allowed to trade (Richardson, 2020).

Already operating with limited cash reserves and small customer bases, small spaza and tuck shops faced severe problems during lockdown and the subsequent price rises (Hull and Masinga, 2023). The government created several financial mechanisms to support small retailers (Crush and Tawodzera, 2024); however, most of the vendors and shop owners included in our study were ineligible for support. To be eligible, one's business had to be formally registered. Foreign nationals lacking adequate documentation were excluded, as were very small, South African‐owned establishments. Many lacked the necessary credit history and sales records required to register or were simply too small to be able to pay taxes as formal enterprises—albeit they contributed to the government's income via value‐added tax payments. COVID‐19 support therefore was a mechanism of government to extend formalisation, excluding many who by necessity operated outside of the formal economy.

### 5.2 Food retail absorbing the shock of COVID‐19 job losses

The collapse of employment in various sectors has led to the establishment of new informal enterprises in food retail. An example from our data yields insight into this process. David, who acquired bricklaying skills in his home country, came to South Africa about 20 years ago to work in construction.^4^ When his employer's company shut down during COVID‐19, he was laid off, and his rental income dried up as tenants faced similar coronavirus‐related losses. His partner's child grants covered only the cost of maize meal and a few extras, leaving the family in a difficult position.

With rising food prices and the loss of his main income source, David needed to find a way to support his family. He started looking for building work but found only occasional, low‐paying jobs. Drawing on his experience of running a tuck shop in his home country, he opened a small outlet from his home, selling sweets, snacks, and later, basic foodstuffs and cosmetics. He sometimes benefited from load‐shedding, as people bought perishable items like meat locally and cooked them the same day owing to unreliable refrigeration. Despite his efforts, David faced hostility from nearby tuck shop owners who were concerned about increased competition in an already saturated market, compounded by corporate retailers from the nearby town.

Rural food systems are rebuilding after the shock of COVID‐19. As the example illustrates, this reconfiguring may entail new networks and entrants from other industries. Many with precarious lines of work lost their livelihoods and are struggling to recover them, while others have been able to create new opportunities.

### 5.3 Multiple shocks

Successive shocks, including COVID‐19, food and fuel price increases, riots, and load‐shedding, had a cumulative, intensifying impact on livelihoods. This is best illustrated through another example. For Mam Ncube, high food and fuel prices expanded the working day and led to exhaustion. Early in the morning, she travelled to farms in Makhathini to buy *imifino* (a green leafy vegetable). From there, she went to town, where she spent the whole day selling the leaves, separated and sold in small bundles. High fuel costs forced up transport prices, reducing her overall earnings each day. Her daily profit was ZAR 80 (about GBP 3.50). She spent her full earnings each day on food for her family.

Like many other informal food system workers, Mam Ncube suffered from exhaustion by working such long days in addition to carrying out the domestic activities necessary to sustain her family. The perishable nature of *imifino* necessitated staying late to sell her stock and repeating the whole routine of traveling to and from the farm each day, rather than selling a larger amount of stock over several days. This increased both the time commitment and transport costs of her work. Her children were mostly grown up (the youngest was 17), but in other similar situations, children's nutrition could be affected when women who travelled far to work and for long hours lacked time to prepare nutritious meals when they returned. In Mam Ncube's case, she would soon lose the child support grant (then ZAR 480 a month), when her youngest child turns 18, a grave loss given the unlikelihood of the young person acquiring employment quickly.^5^

Street vendors and other small and informal food retailers were unevenly affected by the collapse of food supply to the area during COVID‐19 and the July 2021 ‘unrest’. Some male vendors made a sudden profit during the riots as people sought to buy food while supermarkets were closed. However, Mam Ncube's fears for her personal safety deterred her from travelling to town on those days. Consequently, her stock rotted during the period of looting. Personal safety issues also arose during interviews with other women in relation to the rising costs of fuel. Women reported fear of crime because of needing to go to the forest to collect firewood in the context of increasing fuel costs and switching from cooking with gas to cooking on fires (Hull et al., 2023).

For the duration that she was not working, Mam Ncube's family could not buy food and suffered acute hunger. Like many women, she belonged to a *stokvel*. And like many kinds of work that women do, *stokvels* straddle the spheres of production and reproduction. The group collects funds in a shared pool and buys food in bulk to save money. However, load‐shedding has recently caused food to go to ruin because of the sudden lack of refrigeration, leaving large quantities of stored food to rot quickly in the hot summer temperatures. Load‐shedding therefore transforms *stokvels* from a strategy to make money and food go further, to one that is highly risky and potentially wasteful. Furthermore, load‐shedding exacerbated food insecurity in other ways: as well as food rotting in warm weather, refrigerators and other kitchen appliances sometimes broke, imposing a large additional expense on families. Moreover, certain staple foods such as pap (made from maize meal) are spoilt if the cooking process is interrupted.

This story reveals how successive shocks, including COVID‐19, food and fuel price increases, riots, and load‐shedding, have a compounding effect on embodied experiences of hunger, exhaustion, and personal safety risks.

In conclusion, our data show that rural food systems were both ruptured by the COVID‐19 lockdown and subsequent events, while also absorbing some of the shock from other sectors. This produced tensions between informal retailers in a setting where they already faced extreme competition from supermarkets. Labouring under the symptoms of de‐nationalisation, such as globally‐induced price hikes and supermarket dominance, food sector workers also suffered variably depending on their gender, citizenship status, and relative wealth. Punitive police actions towards migrant shop owners, both in relation to our data and in other reported instances around the country, indicate a police force willing at times to mobilise lockdown rules to punish migrants, contributing to processes of re‐nationalisation.

## 6 HUNGER AND STATE LEGITIMACY

After the looting and violence of July 2021 subsided, a national debate took hold about whether the riots indicated intensifying ethnic divisions in the context of xenophobic tensions, or whether they could instead be read as an insurrection against the state, following the traumas of COVID‐19 and rising hunger across the country (Vhumbunu, 2021; Rapanyane, 2022; Enaifoghe, Mtshali, and Durokifa, 2023). Debates often focused attention on KwaZulu‐Natal where the riots began and were most widespread. We argue that singular explanations are inadequate given the range of perspectives offered by our interlocutors, while also noting that xenophobic violence did not occur during the riots in the north of the province, as it did in Durban.

In this section, we review memories and explanations of the riots in uMkhanyakude. Our concern centres on questions of state legitimacy and how this may be linked to the experiences of food and livelihood insecurity set out in the previous sections. We do not treat memories as unmediated descriptions of what took place, even though multiple accounts allow for a tentative understanding. Instead, we recognise that individual memories are informed not only by people's lived experiences of an event at the time, but by subsequent processes of collective memory formation. These in turn are shaped by deeply rooted political subjectivities and by dominant national discourses. Reviewing emerging narratives of the July ‘unrest’, we pivot into a wider discussion on how people from this rural location in KwaZulu‐Natal perceive the state. What are their expectations of the state, and how do they see themselves in relation to it?

### 6.1 The July 2021 riots: a view from uMkhanyakude

One dominant narrative is captured in the concept of the ‘KwaZulu‐Natal problem’. In essence, this pinpoints ethnic allegiances in the province (home to a majority Zulu population) as a major obstacle to building a unified nation. In the recent riots, the racist violence in Phoenix, about 25 kilometres northwest of Durban, was a stark indication of how social unrest can quickly develop along racial lines. Black people there were prevented from entering the suburb and violently attacked, resulting in 36 deaths, primarily from gunshot wounds. These events stirred memories of past racial conflicts, such as the ‘Zulu versus Indian riots’ in 1949, which claimed 142 lives (Ngwane, 2021, p. 314).

In this paper, we have reported examples of xenophobic tensions in relation to the rural food economy in the north of the province. During the riots in uMkhanyakude, a few minor robberies took place at foreign‐owned tuck shops, but there is no evidence that they were targeted for racist or xenophobic reasons. In Jozini, the looting predominantly targeted supermarkets and larger stalls at the shopping mall and in the centre of town. Rioters set alight one supermarket and three others were stripped bare. The shops that were unaffected usually had owners who hired additional security. The only supermarket that was not looted was owned by a local resident as part of a franchise. One interlocutor noted, perhaps accurately, that this owner likely had much greater interest in protecting the store than the large, distantly‐located corporations that owned the others. As the riots subsided, huge queues formed at this single remaining supermarket, with its entrance strictly controlled by security guards. Food prices skyrocketed in the supermarket and at some other smaller stores that had not been looted in the context of severe shortages.

Very few interlocutors revealed that they had participated in the looting themselves. Our conversations suggested that perspectives on the riots and on the motivations of looters varied widely. This raised concerns about relying on singular explanations—whether material, nationalist, populist, or opportunist—for people's involvement in the riots. Explanations that condoned the violence included viewing them as a necessity for feeding families or as a protest against the government, suggesting that rioting was the only way of getting through to the government about local grievances. Food prices were a significant point of complaint and perceived motivation, as well as the new COVID‐19 grant failing to keep up with these prices, indicating profit for supermarkets at the expense of the poor (Hull et al., 2023). Those critical of the riots blamed looters for further price hikes; blame was occasionally levelled at shop owners instead.

Some foreign nationals were deterred by fear of deportation or other punitive police actions. Some of the poorest people in the surrounding area did not or could not loot, often owing to a lack of transport. For example, when the riots broke out, Vusi was travelling home with some food for his family. He was unable to return to town because of the disturbances: ‘I wished I had access to a car on that day because I would have looted enough food for my family’. After the looting had finished, food prices were so high in the shops that remained open that he did not have enough money to buy anything. ‘I thought that my children would die of hunger’, he concluded.

The severity of the food insecurity people faced emerged clearly as a factor in the looting. It is also important to locate these riots in relation to the many other protests in the region in recent years, which have targeted government inaction on water infrastructure and crime (Singh, 2020; Makhaye and Mkhize, 2021). In 2020, the road leading to the nearby government hospital was blocked owing to residents' accusations of nepotistic hiring practices at the hospital (Zincume, 2020). Locating the riots in relation to these other local protests points to deep anger and dissatisfaction with government among a population whose basic needs for food, water, employment, and health are not being met, rather than ethnic mobilisations, being the central issues. One person heard that rioters marched from the mall to a supermarket in the town centre mimicking Zulu infantry of times past, singing struggle songs as they went, before proceeding to loot the supermarket—an account we were not able to verify elsewhere. It is notable that very few people mentioned the Zuma controversy in their reflections about the riots. However, this may not be all that there is to say about the ‘KwaZulu‐Natal problem’. The region's longstanding mixture of competing political affiliations—rather than firm electoral support for the ANC as in most other rural areas—creates a sense of autonomy and non‐loyalty towards government that may affect people's inclination to protest. Several months after the riots, the IFP won a landslide victory in the local elections in Jozini, taking power from the ANC in a seat that typically alternates between the two parties (Makhaye and Mkhize, 2021). This groundswell of support for opposition parties was further confirmed in the national election on 29 May 2024.

### 6.2 Expectations of the state

In interviews, we found a range of perspectives on government, from a strong sense of entitlement to government assistance to a stance of non‐blame towards the state. Extreme food insecurity did not necessarily produce the former sentiment. Let us return to the example of Vusi, which we flesh out in some detail to demonstrate the complexities of political subjectivities as relational and contingent. Vusi was born and grew up in a rural home in the province. He had been working as a street vendor for more than a decade. He lived near to town and was a single father to two children who lived with his mother in their rural home. As well as the income earned from street selling, the family also received support from his mother's pension. They received no child benefits because the documents needed to apply were held with their mothers' families to which he did not have access. His family's food was supplemented by a small quantity of maize and vegetables grown by Vusi's mother, although she was too old to grow as much as she had in the past.

Vusi was forced to stop working when the COVID‐19 lockdown began and, suddenly without income, travelled back to his mother's home: ‘It left me feeling very useless to my children and to my mother back home’. Schools closed immediately in mid‐March owing to the lockdown, but school drivers were still seeking payment for the children's transport at the end of the month. Vusi was relieved when his children's driver did not demand payment, understanding the difficult situation. While at his mother's, he received notice of eviction from the house he had been renting for years.

He managed to return to street selling by slowly building up stock and also started to receive the SRD of ZAR 350 per month. But with increased prices and fewer customers, he was now struggling to pay the basic costs of food, transport, and accommodation. Despite his efforts, his children sometimes went to school without any breakfast, which made him feel like a ‘failing father’.

Reflecting on the role of the government, he said that he did not blame it for his setbacks. Instead, he blamed his parents: ‘They did not send me to school when I was young, but they sent me to look after the livestock all my childhood life and that destroyed my future and hopes’. He was also angry at his father for failing to take care of the family. In contrast, he insisted, the government was doing what it could to help.

In comparison to many accounts given by women, Vusi emphasised his need for self‐sufficiency. This notion of independence was linked to his feelings of dignity as a father. For instance, he mentioned that he thought his mother sought help from neighbours when she ran out of food. But he himself was deeply reluctant to do so: ‘Asking for food from your neighbours is very embarrassing and they gossip about you’. This was reflected in other interviews with men, suggesting a gendered stigma attached to dependence on others.

In this and other interviews, especially those with younger men, desire for self‐sufficiency sat alongside reduced expectations from the state. According to Vusi, the government was doing what it could and was not to blame for his own lack of education or subsequent impoverishment. In another interview, a man who worked as a supermarket trolley porter and received the SRD grant made the following comment when asked about government support: ‘I can't complain too much because I did not even finish school. Maybe if I did finish school, I would be complaining about different opportunities that are not opening to me to go further in life’.

These interviews point to a belief that the government is not responsible for the plight of people lacking formal education. This implied tacit acceptance that possession of formal accreditation is a dividing line between those deserving state recognition and those who do not, affirming the state's role to facilitate upward mobility of deserving citizens into the educated middle classes. Although comments specifically about lack of education were not frequent in the data, they are an example of a wider theme about the desire for self‐sufficiency that is not apparent in the accounts of women. This evokes a longstanding notion among men that self‐worth and respectability are to be achieved by attaining stable employment rather than through mutual help within the community (Hull, 2014, p. 455; Sikweyiya et al., 2022).

We provide this example as a counterpoint to the notion, appearing in debates about riots and the ‘KwaZulu‐Natal problem’, that people can be characterised uniformly as either loyal to or rebellious towards state powers. As people navigate livelihood challenges disturbed by multiple successive crises, their perceptions of the state may be influenced by deeper orientations shaped by gender, age, and relationships, as well as by ethnic identity and political affiliation. Claims on the state are not uniformly articulated, but interact with moral subjectivities based on one's roles, responsibilities, and interpersonal relations.

The July 2021 ‘unrest’ signalled the ineffectiveness of government institutions to withstand pressures from two informal economies: the ‘popular’ economy of the precariat, on the one hand, and the elite network of ANC cadres, blamed for igniting the protests in defence of the Zuma‐led faction inside an embattled ANC, on the other. These two are connected. They are both dramatic expressions of capitalism's inherent contradictions, its tendency to destabilise the very social and political systems it depends on for long‐term accumulation (Fraser, 2015). The causes of the riots cannot be reduced only to the re‐nationalising rhetoric of Zulu nationalism or xenophobia; they also encompass the symptoms of de‐nationalisation, including widespread joblessness and food insecurity. Hunger is contributing to a legitimation crisis for the ANC, but this was also well under way, indicated by longstanding dissatisfaction and ongoing protests. Political non‐allegiance to the ANC is evident in election results, yet sentiments towards the state interact with personal life histories and relationships to produce varying expectations that defy singular explanation.

## 7 CONCLUSION

This paper has explored the politics of food security in South Africa through the theoretical frameworks of de‐ and re‐nationalisation (Hart, 2014). By tracing their manifestations and effects in the rural food economy, we have highlighted how these contradictory processes exacerbate food insecurity and political instability. They create the political terrain in which food security demands on the state are understood and articulated, referred to by Patta Scott‐Villiers and Naomi Hossain (2017, p. 2) as the ‘politics of provisions’.

De‐nationalisation in South Africa has manifested through a neoliberal economic model that benefits global market interests, rendering the country's food system highly vulnerable to international market fluctuations. This results in a food economy in which people eke out a living in volatile and competitive conditions, compounded by events including the COVID‐19 lockdown and the price hikes. The rural food economy's capacity to absorb and mitigate shocks was compromised by a confluence of factors including governmental action and inaction, world price fluctuations, and localised economic pressures.

In contrast, re‐nationalisation has resurfaced in the form of populist and xenophobic rhetoric, reflecting an attempt by political actors to consolidate support through nationalist appeals and anti‐immigrant sentiments. This has led to increased tensions in the food sector, particularly affecting foreign‐owned businesses. We identified these in our research in northern KwaZulu‐Natal, the province known as the epicentre of the 2021 riots. The uneven distribution and accessibility of state support negatively affected those with precarious livelihoods and undocumented status. Migrants faced severe hardships owing to their exclusion from government assistance during the lockdown. This situation was worsened by rising xenophobia and fears of deportation. Yet, our analysis suggests that the riots, often interpreted through the lenses of ethnic division and xenophobic aggression, were more closely linked to broader issues of food insecurity and governmental dysfunction in uMkhanyakude.

Furthermore, our analysis reveals how perceptions of the state are shaped by individual and collective experiences of food access and livelihood challenges, as well as by personal histories, gender roles, and interpersonal relations. While some view the government critically, others express a blend of acceptance and self‐reliance. Nevertheless, while the causes of the July 2021 riots were diverse, they should be seen as part of a larger pattern of discontent linked to the systemic forces of de‐ and re‐nationalisation. Food insecurity is an outcome of these processes while also contributing to a deepening legitimation crisis for the state as South Africa faces an uncertain political future.

## CONFLICT OF INTEREST STATEMENT

There are no conflicts of interest to report.

## ETHICS STATEMENT

This paper reports analysis of primary data. The ethics of data collection and analysis were approved by the SOAS Research Ethics Panel. Data have been kept confidential and used anonymously.

## FUNDING STATEMENT

The research was funded by the SOAS Research and Knowledge Exchange fund.

## Data Availability

Research data are not shared.

## Endnotes

^1^
Since the beginning of the COVID‐19 pandemic in March 2020 until September 2023, there was an increase of about 35 per cent in the price of food (Fourie, 2023).

^2^
Consumer food price inflation accelerated rapidly to 14.0 per cent in February 2023 (Pettifor, 2023).

^3^
The ANC won 40 per cent of the national vote in the 2024 election. The results can be viewed at the following website: https://results.elections.org.za/home/ (last accessed on 3 October 2024).

^4^
All names of interlocutors are pseudonyms.

^5^
The child support grant increased to ZAR 520 per month on 1 April 2024.
